# Robust Algorithms for Drone-Assisted Monitoring of Big Animals in Harsh Conditions of Siberian Winter Forests: Recovery of European elk (*Alces alces*) in Salair Mountains

**DOI:** 10.3390/ani12121483

**Published:** 2022-06-08

**Authors:** Alexander Prosekov, Anna Vesnina, Victor Atuchin, Aleksandr Kuznetsov

**Affiliations:** 1 Laboratory of Biocatalysis, Kemerovo State University, 650043 Kemerovo, Russia; aprosekov@rambler.ru; 2 Laboratory of Natural Nutraceuticals Biotesting, Research Department, Kemerovo State University, 650043 Kemerovo, Russia; koledockop1@mail.ru; 3 Laboratory of Optical Materials and Structures, Institute of Semiconductor Physics, 630090 Novosibirsk, Russia; 4 Research and Development Department, Kemerovo State University, 650000 Kemerovo, Russia; 5 Department of Applied Physics, Novosibirsk State University, 630090 Novosibirsk, Russia; 6 Department of Industrial Machinery Design, Novosibirsk State Technical University, 630073 Novosibirsk, Russia; 7 Computer Engineering Center, Kemerovo State University, 650043 Kemerovo, Russia; adkuz@inbox.ru

**Keywords:** large animals, computer technology, UAV, comparison of accounting methods, nature reserve

## Abstract

**Simple Summary:**

Forest animals can be used as a sensitive indicator of the real state of biodiversity. The research objective was to study the potential of drone planes equipped with thermal infrared imaging cameras for large animal monitoring in the conditions of Siberian winter forests with snow background at temperatures of −5 °C to −30 °C. The surveyed territory included the Salair State Nature Reserve in the Kemerovo Region, Russia. Drone planes were effective in covering large areas, while thermal infrared cameras provided accurate information in the harsh winter conditions of Siberia. The research featured the population of the European elk (*Alces alces*), which is gradually deteriorating due to poaching and deforestation. The designed technical methods and analytic algorithms are cost-efficient and they can be applied for monitoring large areas of Siberian, Canadian and Alaskan winter forests.

**Abstract:**

There are two main reasons for monitoring the population of forest animals. First, regular surveys reveal the real state of biodiversity. Second, they guarantee a prompt response to any negative environmental factor that affects the animal population and make it possible to eliminate the threat before any permanent damage is done. The research objective was to study the potential of drone planes equipped with thermal infrared imaging cameras for large animal monitoring in the conditions of Siberian winter forests with snow background at temperatures −5 °C to −30 °C. The surveyed territory included the Salair State Nature Reserve in the Kemerovo Region, Russia. Drone planes were effective in covering large areas, while thermal infrared cameras provided accurate statistics in the harsh winter conditions of Siberia. The research featured the population of the European elk (*Alces alces*), which is gradually deteriorating due to poaching and deforestation. The authors developed an effective methodology for processing the data obtained from drone-mounted thermal infrared cameras. The research provided reliable results concerning the changes in the elk population on the territory in question. The use of drone planes proved an effective means of ungulate animal surveying in snow-covered winter forests. The designed technical methods and analytic algorithms are cost-efficient and they can be applied for monitoring large areas of Siberian and Canadian winter forests.

## 1. Introduction

The expanding human population increases the chance of human contact with nature, which inevitably reduces or changes the natural habitat of wild fauna [1,2,3,4]. Mining and industrial use of pristine lands, as well as urban and rural development, produce a human-induced impact on the environment, thus causing the transformation and degradation of natural biocenoses and increasing/reducing biodiversity [5,6]. In the conditions of limited natural resources, the effectiveness of environmental management decisions depends on an accurate and timely analysis of environmental data. Therefore, improved methods of information collecting and processing can lower the environmental impact of managerial actions in the sphere of rational exploitation of natural resources and natural balance [7,8,9,10,11]. A consistent approach to environmental management requires new systems of effective environmental monitoring. Surveys of large mammal population and distribution remain an urgent task. Representatives of the local fauna are among the most significant markers of the state of the environment and they are sensitive to its changes [12,13].

Animal surveys contribute to the rational conservation of biodiversity. A proper analysis of statistics on migration, fertility and mortality can reveal cases of poaching and assess its real scale [3,4,6,10,11,14]. A competent approach provides basic data for informed managerial decisions on the matters of animal population as an integral part of national wealth [15,16,17,18,19]. Animal survey is an important control factor that helps to balance socio-economic and natural interests. The existing methods are based on direct counting or indirect evidence, e.g., footprints, feces, etc., as their results often prove unreliable [10,14,17,20,21]. Based on old databases, these methods are expensive and time-consuming [22]. Moreover, most traditional methods require direct participation of humans, who cannot work systematically under the harsh conditions of Siberian winter forests and can affect the life of animals [23]. The aerial survey method is an exception as it presupposes direct observation of animals from an aircraft.

Digital technologies are the most promising way to improve traditional survey methods of land and air accounting. They can reduce the shortcomings of manual methods and simplify their implementation. This approach is especially promising for monitoring the environment in the vast and sparsely populated forests of Siberia. For example, the use of a GPS system makes it easier to determine the length of the daily tracks left in snow by a wild mammal [24]. Winter track count is a typical animal survey method in Russia [25]. GPS collars make it possible to monitor the life of endangered animals, e.g., Amur tigers, cougars, brown bears, etc. [26,27,28,29,30,31,32]. Trail cameras are another positive example of digital technology in this sphere [9,33,34].

Nowadays, aerial accounting often requires the use of drone planes and copters equipped with various sensors and cameras [35,36,37,38,39,40,41,42,43,44,45,46]. Unmanned aerial vehicles (UAVs) equipped with radio receivers can track the routes of animals with GPS collars, i.e., as a biotelemetry method [47], for sampling [48], for collecting data from a particular habitat [49], and in anti-poaching operations [35]. N. Das [50] used UAVs to monitor and collect data on terrestrial and aquatic bird species. C.N. Scholten [23] used a UAV with a thermal imager to locate nests of songbirds. The work noted that the use of UAVs is less destructive in comparison with the traditional method of accounting (counting animals). In the work of L.F. Gonzalez [22], a UAV with thermal imagers was used to detect wild animals; the need for automatic processing of the received data was reflected. The use of UAV eliminates any possible threat to the operator and the researcher team. In addition, UAVs produce low noise pollution, thus increasing the reliability of the survey. They can cover large remote areas in a short time. However, the use of UAVs for animal surveys requires reliable algorithms that would allow naturalists to obtain the necessary information with minimal impact on animal behavior [37,51,52].

In dense forests, however, camera-based visual counting is almost impossible, if large areas should be observed in real-time mode. Modern thermal-imaging systems can solve this problem. They provide high noise immunity even in complex environments [39,40,53,54]. However, UAVs require modern software to process the bulk of high-resolution real-time video they record during each flight [55]. In the present work, the research objective was to develop and test effective methods for monitoring the population of large warm-blooded animals in the winter conditions of Siberian forests. The areas in question are large and sparsely populated, which makes it difficult to control the current state of the environment using traditional methods. The Siberian winter lasts 4–6 months, depending on the latitude. The snow cover is total while the temperature drops below −55 °C from time to time. To survive in the harsh conditions of low temperatures and limited food resources, forest species seek salvation in long hibernation. European elks (*Alces alces*), grey wolves (*Canis lupus*), and Siberian roe deer (*Capreolus pygargus*) are almost the only large warm-blooded forest species of Siberia that do not hibernate. The present study featured the European elk as a test object for the animal survey. With its weight reaching ~600 kg, it is the largest deer species on the planet (Figure S1). The experimental survey covered the uninhabited territory of the Salair Nature Reserve, located in the northern part of the Salair Ridge (Kemerovo region, Russia) near the Tanay ski resort (54°42′46″ N, 85°3′42″ E).

## 2. Methods

Commonly, to estimate elk population in Kemerovo region, the daily track count is implemented in the range from 1 January to 28 to 29 February. In 2019 and 2020, this work was made by 27–28 February and 28–29 February, respectively. On the first day, foresters and volunteers filled up the existing tracks with snow. On the second day, new tracks were counted. The method of winter tracking is described in more detail in the Methodology for Accounting the Number of Hunting Resources by the Method of Winter Route Accounting [56]. Parallel to the track count, they used drone-mounted thermal infrared cameras: on 26 February 2019 and 29 February 2020. The drone planes were Supercam S250 (Unmanned Systems LLC, Izhevsk, Russia). Table 1 demonstrates the characteristics of this model. Its take-off weight is 7.5–9.5 kg, which allows for 1.5 kg of payload, e.g., a camera and a thermal imager.

The drone plane can operate at wind velocity of up to 15 m/s and air temperature from −50 °C to +45 °C. In addition, it can withstand moderate rain or snowfall. These advantageous characteristics make it possible to monitor the territory in almost any weather conditions. The drone plane carries a receiver of the global satellite navigation system (GNSS) for precise coordinate control and positioning of photography points. It has the capabilities of bungee launch and parachute recovery.

Table 2 and Table 3 show the main characteristics of the camera and the thermal imager, which is important for understanding the capabilities and limitations of this study. Sony RX1R II is a full-frame camera with no crop factor, which makes it possible to cover a wide area without additional maneuvering.

The camera is compact: in fact, it is one of the smallest full-size cameras and weighs less than 500 g. The lens does not have to be changed. In addition, it is one of the cheapest cameras in its class. The high resolution allows for visual identification of various animal species in the photos and video. We chose a compact, light, and low-power ATOM500 (weight 32 g) thermal-imaging camera. Its allowable range of working conditions is quite impressive: this camera can be used in extreme temperature and humidity conditions. As assumed, the sensitivity level allows it to identify thermal signatures of animals against the underlying surface even at temperatures below −10 °C, i.e., for much of the year. In winter, the European elk is covered by thick fur and, respectively, the fur surface temperature drastically lowers the temperature of the body (35.8–37 °C). For this reason, the temperature difference between fur and snow surfaces is unclear, and the detection of thermal anomaly of the European elk on the snow background is not a trivial task. Thermal imaging is able to detect animals by their thermal signature according to the contrast between the body temperature and the environment, which might reach 30–40 °C. Therefore, winter surveys are more efficient. Unfortunately, the method cannot tell the difference between species of similar mass and shape, e.g., a wolf and a wild boar.

**Figure 1:**
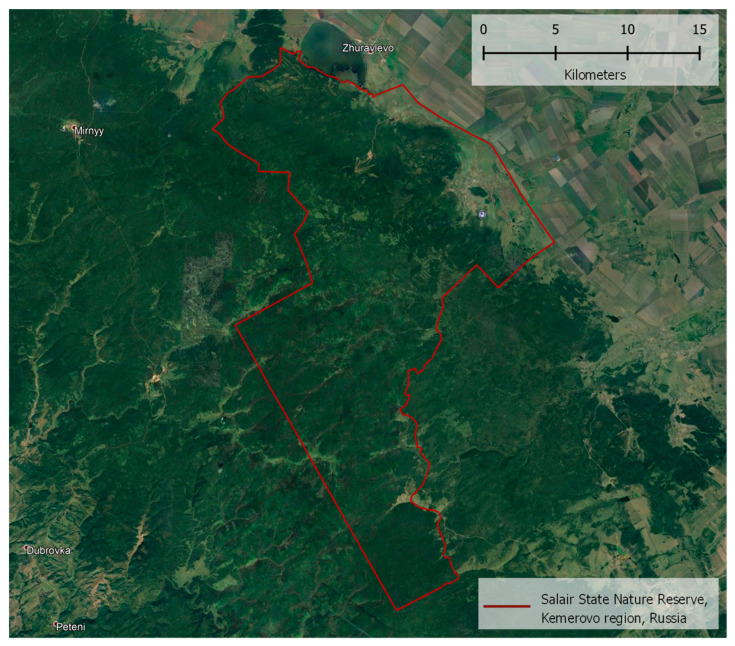
The red contour is the territory of the Salair State Nature Reserve, Kemerovo region, Russia, Asia.

The research covered the territory of the Salair Nature Reserve (Kemerovo region, Russia). The study was organized in this area, since the Salair State Nature Reserve is a habitat for a large number of elk in winter, compared to other nearby areas. The reserve was created in 2000 as a species reserve for the protection and reproduction of elk. The reserve is an environmentally sensitive territory of regional significance. Figure 1 specifies its geographical location.

The Salair national park is mostly black taiga of firs and aspens with patches of birch and aspen undergrowth. The elk is one of the main protected species in the park. Therefore, its population survey is an important tool of its protection and reproduction [57,58]. The Salair taiga borders on agricultural steppe areas in the east, north, and west. In the south, it joins the taiga massif of Gornaya Shoriya and Altai.

The drone planes delivered a large volume of photo and thermal imaging. We processed the obtained data using the Thermal Infrared Object Finder (TIOF) software developed at Kemerovo State University. The application was designed in Python and can be installed on any computer. It is capable of processing a large amount of infrared image data to identify specific animals. The analysis fixes the so-called thermal anomalies, which are warmer than the ambient temperature and indicate the presence of an animal [59]. To determine the effectiveness of the developed algorithm, we compared the UAV survey results with those obtained by the traditional daily track count in 2019–2020. Technical details are given in Table 4.

## 3. Results and Discussion

Visual analysis of conventional photo and video made it possible to identify animals with the same thermal signature. The simultaneous use of photo and thermal imaging improves the accuracy and reliability of aerial surveys. Figure S2 provides an example of such an analysis. The left image shows three types of objects: the white of the snow background, the numerous translucent round crowns of naked trees and shrubs, and the dark round crowns of coniferous trees. Under this resolution, the patchy background of winter taiga makes it hard to detect heat signatures in the photo image: the silhouettes of trees and shrubs obscure the contours of the animals. However, the thermal image on the right clearly shows the signatures of two elks as their body temperature differs significantly from the fairly uniform temperature background of the winter taiga.

In 2019, it took two flights to survey the territory. The pictures were taken from altitude 250 ± 10 m. Figure S3a shows the flight routes. The dots indicate the centers where RGB images were taken. In 2020, we launched one flight, its route is shown in Figure S3b. To facilitate the comparison, we placed a fan shape of glades into the bottom left corner of both Figure S3a,b. The glades are the system of ski slopes of the Tanay ski resort. Table 4 demonstrates the basic technical information on the flights.

We developed the following algorithm to process the images obtained from the drone planes:

(1) We sequenced the infrared video with an interval of ~0.6 s.
(2) After that, the infrared images were processed using software according to the degree of color intensity and pixel clusters. As a result, we obtained numerous infrared images with thermal extremes, which indicated an object with a higher temperature than that of the snow, e.g., an animal, a human, or a car.
(3) We uploaded the RGB photos and telemetry into the Agisoft Metashape Professional software for alignment.
(4) The infrared images underwent a visual inspection for the initial screening of “junk” data.
(5) The coordinates of the infrared images with extremes were compared in-camera with the aligned RGB photographs, and the presence of large game was determined visually.
(6) Finally, we compared the research results at different stages.

**Figure 2:**
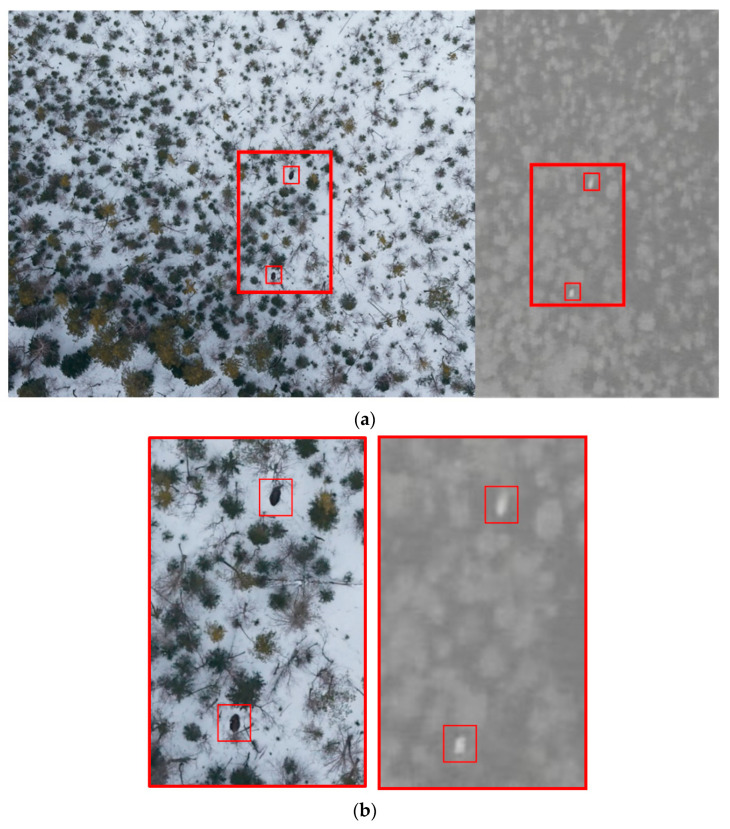
RGB vs. infrared images at (**a**) low and (**b**) high resolution.

Figure 2 gives an example of comparing images in the visible and IR spectra. The low-resolution infrared image (Figure 2a, right) shows two thermal signatures. However, the photo image with a similar resolution (Figure 2a, left) provides no reliable identification of the signatures. When the resolution was increased, the body contours of two elks became visible—see the red frame in the photo image (Figure 2b, left).

The analysis employed software developed by the Kemerovo State University which allows *jpeg* and *png* image processing. The processing time depended on the number of images: it took the program 25–50 s to process materials of one standard UAV flight that lasted 100–150 min. The software allows for a thermal sensitivity that exceeds the capabilities of a human observer. Taking into consideration the limited flight time, this made it possible to detect even weak thermal anomalies. Figure S4 gives a comparative analysis of the processed results for infrared images taken from a height of 200 m and 400 m. Figure S4 shows a thermal signature that is clearly visible to the human eye. The shot was made from a height of 200 m. When the same area was shot from 400 m, the same thermal signature was almost indistinguishable to the human eye, while the software application was able to detect it.

**Figure 3:**
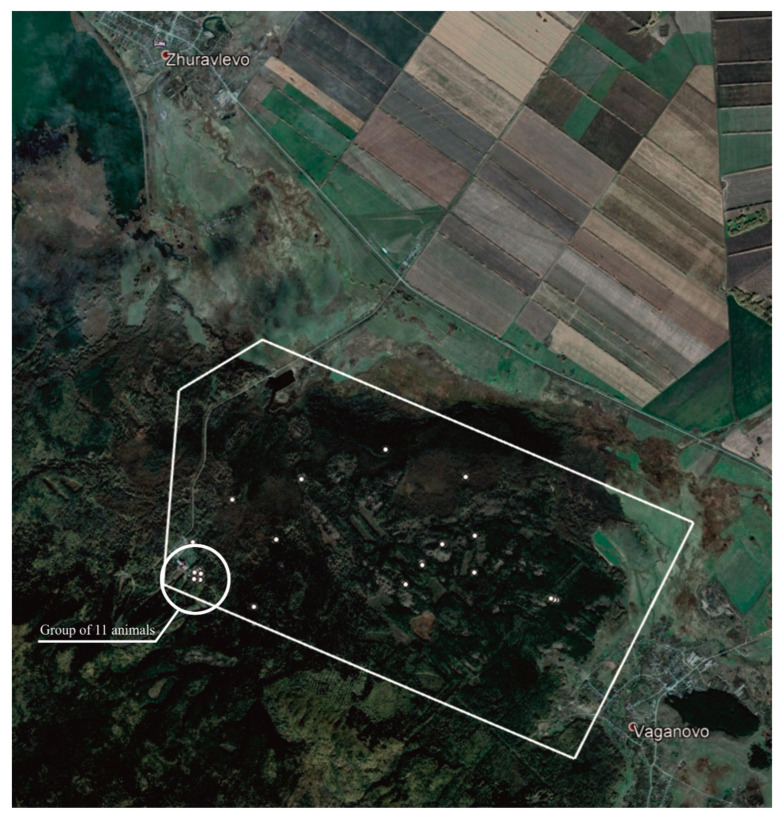
Map of the elk population in the area surveyed in 2019. A cluster of animals was detected on the territory of the rehabilitation center of the Tanay ski resort.

The survey of 2019 detected 34 objects (numbers 1–34). Figure 3 shows their spatial distribution.

Out of 34 objects, numbers 1–25 are elks. Table S1 shows the coordinates of the animals detected by the drone planes in 2019. The detected objects (34) also included untargeted objects not related to wild animals, e.g., a human person and a group of animals contained in the rehabilitation center of the Tanay ski resort.

**Figure 4:**
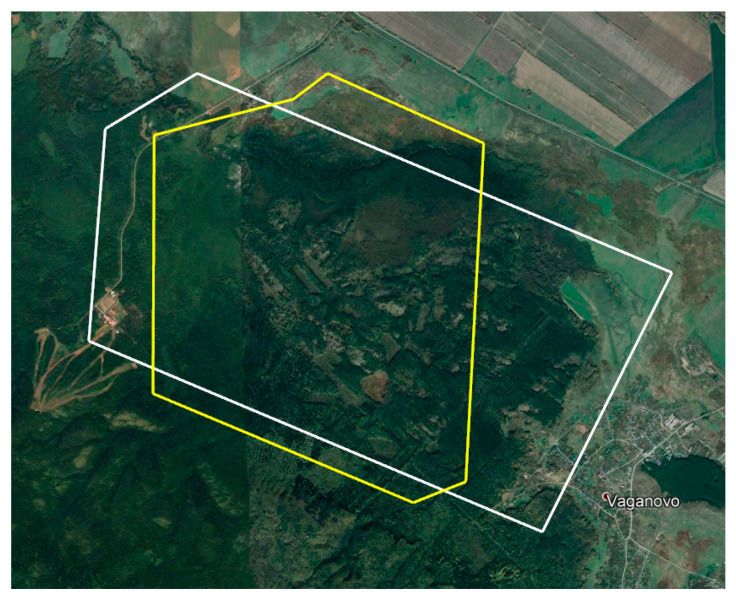
The boundaries of the territories surveyed in 2019 and 2020.

The Tanay resort caused too many “false positives”. As a result, the contour of the scanning section had to be changed in 2020 to exclude the Tanay resort premises. Figure 4 demonstrates the ratio of the scanned areas in 2019 and 2020.

**Figure 5:**
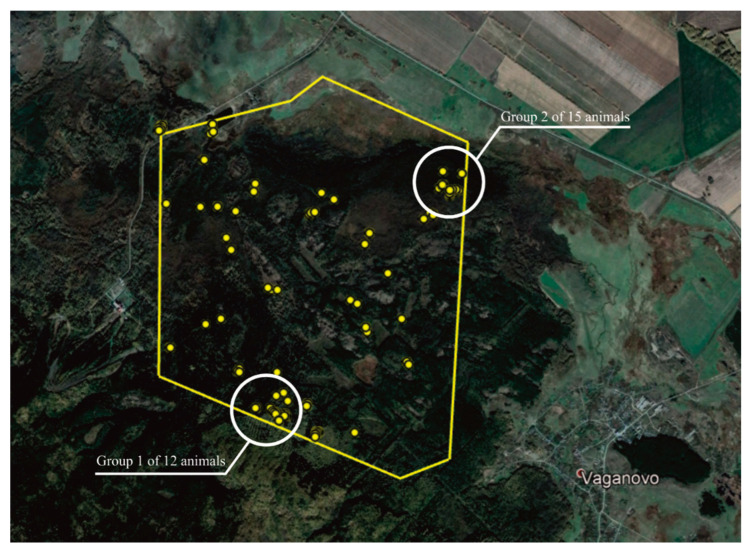
Map of the elk population in the area surveyed in 2020. Two animal clusters were identified outside the Tanay resort.

The survey of 2020 revealed 63 objects, of which 55 were elks. Figure 5 shows their spatial distribution.

Table S2 specifies the coordinates of the animals detected by the UAV survey in 2020. We failed to calculate the coordinates of numbers 20, 21, and 22 on the RGB images as these objects were too close to the frame. The remaining objects (8) were people. Figure S5 demonstrates a test snapshot of untargeted search objects—some random fishermen that happened to be in the area.

**Figure 6:**
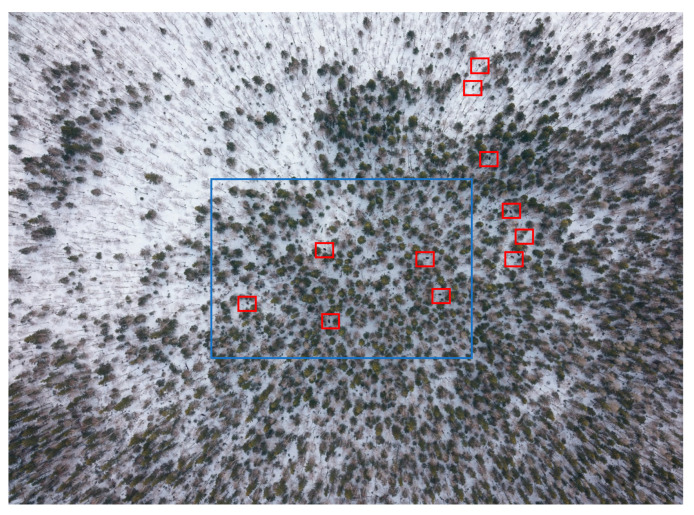
RGB image of Group 2.

The map of elk distribution (Figure 5) shows two clusters, the largest one being Group 2, which included 15 elks. Figure 6 demonstrates the maximum number of animals recorded in one RGB image—11 elks.

**Figure 7:**
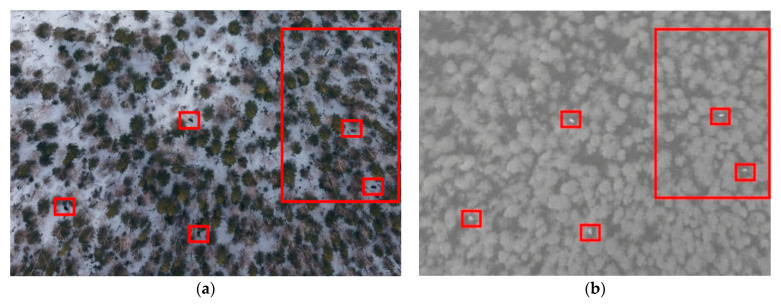

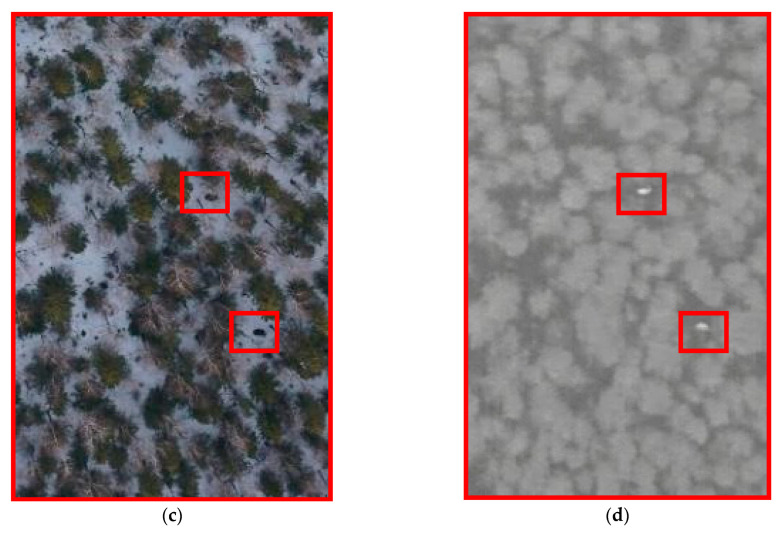
Comparison of RGB and infrared images of Group 2: (**a**) part of the RGB image corresponding to the capture of the infrared image; (**b**) the infrared image corresponding to image (**a**, **c**) and (**d**) are zoomed-in images of (**a**, **b**), respectively.

The maximum number of animals fixed in one infrared image was five elks (Figure 7).

This difference resulted from the different technical characteristics of the thermal infrared camera and the visible spectrum equipment. The bandwidth of the infrared image was 1/3 in the center of the width of the visible spectrum. In Figure 6, the outline of the infrared image is blue. Thus, the shooting area of the infrared image was approximately nine times smaller than the shooting area of the RGB image. All the routes were planned specifically to achieve a transverse overlap of 10–15% for infrared imaging, in which case the overlap of RGB images was 70%. Figure 7 compares RGB and infrared images of the same surface areas in Group 2. It becomes clear that the distance between the elks was about 50 m.

**Figure 8:**
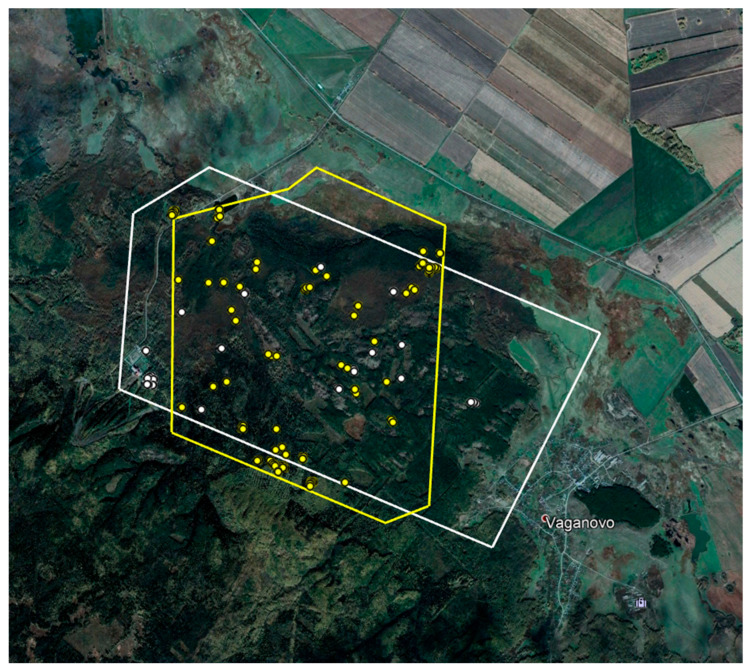
The area under survey and the distribution of elks in 2019 (white) 2020 (yellow).

A comparative analysis of the data obtained in 2019 and 2020 revealed that 25 and 55 elks were identified in 2019 and 2020, respectively, where the two studied areas overlapped (15.9 km^2^). Therefore, the number of animals within the same habitat almost doubled. Figure 8 shows their spatial distribution.

The survey of 2020 revealed two clusters of elks. Such uneven distribution could be explained by some behavioral characteristics of the animals. We detected two wolves during the visual analysis of the images obtained in 2020 and the corresponding infrared images with thermal anomalies over an area of 7 km^2^ (Figure S6). It was the first time wolves had been detected in the Salair Nature Reserve. No traditional track counts had ever revealed wolves in this territory, and naturalists had always considered the park a wolf-free zone. According to daily track counts, the wolf population had almost disappeared in the Kemerovo region by 2015–2017 as a result of man-induced factors: rangers reported only accidental visits from the neighboring regions [60,61]. Thus, the developed method of a digital survey provided a more complete identification of large animals in the given habitat. Accuracy is especially important for monitoring the population of such large predators as wolves. Mistakes can have an extremely negative effect on the managing populations of herbivores.

During the aerial surveys of 2019–2020, the population, distribution, and habitat of the European elk proved to correspond to the data obtained by the traditional method of winter track counts submitted by the Department of Animal Object Protection of the Kemerovo Region. Presented data from the Department: the approximate number of elk in the territory in 2019 is 30 ± 6 individuals and in 2020 is 50 ± 7 individuals. The data are not precise due to the limitations of the method. We registered a significant increase in the elk population in the forests of the Salair Ridge. In addition, we detected wolves in the surveyed area. The research justified the combined use of various digital technologies for game animal survey, i.e., photo and thermal imaging. The equipment performance was good even in the harsh winter conditions, which means great prospects for research on larger areas.

## 4. Conclusions

The current state of Siberian forestry requires new methods of the environment control and competent resource management. Therefore, traditional methods for animal surveying have to be perfected. Digital technologies proved to be the most promising method that can improve the shortcomings of traditional accounting methods. These technologies eliminate the problem of inaccessibility of research sites and reduce error probability caused by the human factor. According to the new methodology, the survey was carried out automatically using drone-mounted thermal infrared cameras, and the data processing was performed by specialized software.

This research featured the population of the European elk (*Alces alces*) in the territory of the Salair Nature Reserve using drone planes with two types of payload. The obtained data on the elk population confirmed the results obtained by the traditional winter track counts. This indicates that:

(1) Aerial surveys are a promising practical method for determining the population of large ungulate animals, e.g., elks, roe deer, wild boars (*Sus scrofa*), red deer, as well as wolves.
(2) Drone-mounted thermal infrared cameras provide accurate data on the animal presence in the winter period. The combined use of RGB images and thermal-imaging cameras allows for reliable identification of the thermal signature of the detected object.
(3) The method can be used to check the data obtained by traditional survey methods, i.e., as a part of a complex survey.
(4) Unmanned aerial vehicles make it possible to monitor vast forest areas in a short period of time. This advantage allows scientists to observe animal behavior in winter.

## Figures and Tables

**Table 1:** Specifications of UAV Supercam S250.

Characteristic	Description
Wingspread	2.5 m
Flight time	3 h
Flying range	≤180 km
Engine	Electric
Radio line range of action	50–70 km
Lift flight	50–500 m
Velocity	65–120 km/h
Working flight altitude	150–5000 m

**Table 2:** Specifications of Sony RX1R II camera.

Characteristic	Description
Matrix	Full-frame Exmor R^®^ CMOS sensor
Resolution/Pixel size	35.9 × 24.0 mm/35 mm full frame
Screen format	3:2
Resolution	About 42.5 MP
ISO	100–25,600 (1/3 EV steps)

**Table 3:** Specifications of thermal-imaging module ATOM M500.

Characteristic	Description
Type of infrared receiver	Uncooled microbolometric amorphous silicon matrix
Resolution/pixel Size	640 × 480/17 µm
Sensitivity	≤60 µm at 300 K with a F#1.0 lens
Frames per second	50 hz
Spectral range	8~14 µm

**Table 4:** Data on UAV flights.

Duration of one flight	2.5–3 h
Flight speed	70–100 km/h
RGB camera frame capture width/length	257/171 m
The distance between the centers of photographing (frequency of shots)	34 m
Coverage area for one flight	~6 km^2^
Number of images per flight	~3500 images
Width/length of capture of the frame of the thermal-imaging camera	78/58 m
The number of thermal-imaging images obtained during video storyboarding	~240,000 frames
The thermal imager shot in the continuous video stream mode at a frequency of 25 frames per second. Furthermore, the storyboarding and processing of these frames as separate photographic images was carried out	

## Data Availability

Data related to this work are available from authors.

## Supplementary Materials

The following supporting information can be downloaded at: https://www.mdpi.com/article/10.3390/ani12121483/s1, Figure S1: Photo of young elk contained in the rehabilitation center of the Tanay ski resort; Figure S2: Photo (left) and thermal (right) imaging of the same area. The red frame marks the spot with thermal signatures of two elks; Figure S3: Flight routes in 2019 (a) and 2020 (b) on a Google map (Salair State Natural Park); Figure S4: Infrared images processed by Thermal Infrared Object Finder: (a) height of exposure is 200 m, (b) height of exposure is 400 m; Figure S5: An example of untargeted objects (fishermen); Figure S6: Snapshot where visual inspection revealed two wolves (red circles at the bottom) and an elk (red circle at the top); Table S1: Coordinates of elks detected in 2019 (WGS 84); Table S2: Coordinates of elks detected in 2020 (WGS 84).
