# Supplementary material for: Robust Algorithms for Drone-Assisted Monitoring of Big Animals in Harsh Conditions of Siberian Winter Forests: Recovery of European elk (Alces alces) in Salair Mountains
Source: Animals (Basel). 2022 Jun 8;12(12):1483. doi: 10.3390/ani12121483 (PMC9219499; doi:10.3390/ani12121483)
Supplement: Supplementary file 1 [file animals-12-01483-s001.zip › animals-1701363-supplementary.pdf]

Supplementary Information

Recovery of European elk (*Alces alces*) in Salair mountains, South-West Siberia: Drone-assisted monitoring in Siberian winter forests

Alexander Prosekov[1], Anna Vesnina[2], Victor Atuchin[3,4,5,6], Alexander Kuznetsov[7]

[1]Laboratory of Biocatalysis, Kemerovo State University, Kemerovo 650043, Russia

[2]Laboratory of Natural Nutraceuticals Biotesting, Research Department, Kemerovo State University, Kemerovo 650043, Russia

[3]Laboratory of Optical Materials and Structures, Institute of Semiconductor Physics, Novosibirsk 630090, Russia

[4]Research and Development Department, Kemerovo State University, Kemerovo 650000, Russia

[5]Department of Applied Physics, Novosibirsk State University, Novosibirsk 630090, Russia

[6]Department of Industrial Machinery Design, Novosibirsk State Technical University, Novosibirsk 630073, Russia

[7]Computer Engineering Center, Kemerovo State University, Kemerovo 650043, Russia

Table S1. Coordinates of elks detected in 2019 (WGS 84)

№	Longitude	Latitude	Height
1	85.110297848359806	54.730882219372198	198.98561096243401
2	85.127968663350799	54.727463169289997	201.316118139271
3	85.1279980885366	54.727410639217098	204.71284894195301
4	85.091813130997821	54.72730194701019	222.55680083617261
5	85.091905888007929	54.727184156672017	225.3753855794611
6	85.091708557300862	54.727100148412021	224.34627755492613
7	85.130110157872863	54.719981220366549	220.54264831474447
8	85.114585161373597	54.713958210657403	242.43547057963599
9	85.114948278473904	54.713809023850096	244.79338073692799
10	85.147677349246706	54.711876080208903	230.990292456194
11	85.147627794512701	54.712015259151698	242.92978503012699
12	85.146957292600504	54.712022614058199	231.627746636458
13	85.130004854285701	54.715318525401102	218.78112793009899
14	85.123209076298494	54.718970809625702	237.70294189545899
15	85.122961963739897	54.718898380683903	239.56391906777199
16	85.118536502137204	54.716249879510997	221.517395018672
17	85.118399593548105	54.716554808522297	234.57287597599699
18	85.085931524622595	54.719452890135301	208.32406616112701
19	85.086188411933094	54.719458618632302	208.78538513122001
20	85.081389309516396	54.710931295882098	227.650070190695
21	85.068577473814997	54.714423646681198	232.35325318607201
22	85.068414405093307	54.714452562385702	231.099900811651
23	85.068245312156506	54.714444303126903	234.458126994699
24	85.0764515110247	54.724528622543801	204.60606384154801
25	85.148245606787597	54.711821588297603	237.38904026108401

Table S2. Coordinates of game animals detected in 2020 (WGS 84)

№	Longitude	Latitude	Height
1	85.118772012433098	54.713804978679498	221.24926563737901
2	85.118837676545596	54.7138030567803	220.96646109948199
3	85.1184037538588	54.724069837266597	161.53108932726099
4	85.127608183541099	54.709537661661699	205.73968113507101
5	85.127578848055606	54.709544409565602	205.189070817735
6	85.128024611689597	54.709186571973497	222.54406642907099
7	85.111727056238095	54.729591752622099	158.97178500281601
8	85.109044347298294	54.730412572146697	159.902602220966
9	85.108308574505301	54.700672574516801	233.04602847664199
10	85.108038792392307	54.700618908346698	210.82084875381901
11	85.107973982070405	54.700559080735303	211.11285263881999
12	85.108558386140302	54.700761272005103	232.752580996982
13	85.108472270559503	54.700863059203002	209.247098181572
14	85.108535177148397	54.700874286785499	232.918694260367
15	85.108173944998299	54.700919420003999	209.262489730309
16	85.108254122142597	54.700905563592499	209.158620436684
17	85.108239047667695	54.700951086982599	209.201580218662
18	85.108608116665707	54.700950559150499	229.50630024322899
19	85.108801776431804	54.700843659697902	232.36783291868201
20			
21			
22			
23	85.107975040701007	54.700263020098198	233.82243981525599
24	85.106074624602002	54.704055701628398	210.658225858972
25	85.106136573544006	54.7040781110612	208.506290579926
26	85.099493050048096	54.7053079845833	194.693390633955
27	85.091518701712005	54.708658630669099	188.366825762454
28	85.091530339948307	54.708224454460797	199.64649418043001
29	85.091101266579997	54.7083131361656	198.346416968038
30	85.088544676305503	54.724835223797797	161.866297719062
31	85.083764188331998	54.734475179515599	156.84730392176999
32	85.083460244367899	54.734490474254997	159.06984489701199
33	85.084187704105304	54.7141389614807	175.10283153987299
34	85.076480257111101	54.711266149186301	184.894166058671
35	85.076678184294707	54.711247381929098	184.31203705996501
36	85.136639988474897	54.7302353404786	172.98087417473499
37	85.136697253107101	54.730052406213801	161.67201879267299
38	85.136718433450298	54.730355708985499	168.238824877008
39	85.136656271645904	54.7303827015241	168.833962021921
40	85.136656596209804	54.7305851193899	166.104771995058
41	85.136710685780301	54.730767016744998	165.17702759190399
42	85.137287276462899	54.730688414516898	167.50129595140601
43	85.137975306157898	54.730567994329398	166.769509816631
44	85.137256106620001	54.730852065401997	162.894958685586

45	85.138599453311002	54.730713208617402	163.58592180608599
46	85.138054663839398	54.730847958134802	166.93364169922501
47	85.139383419927299	54.732827205225597	175.79791899629501
48	85.135112799721696	54.7314238837345	162.491229883404
49	85.135347320606499	54.731417515108802	170.50014156005801
50	85.135033323570298	54.731276818987801	164.74014906093001
51	85.134345782434707	54.730985132185999	164.96353774501799
52	85.1352233461254	54.733086959395202	171.264460928955
53	85.136678509299415	54.730763479449031	171.37374554407111
54	85.139316704188374	54.732814909614206	170.16974337188446
55	85.135344734301498	54.731472021594357	170.46117875363277

Coordinates 20-22 were not calculated because the objects were too close to the frame.

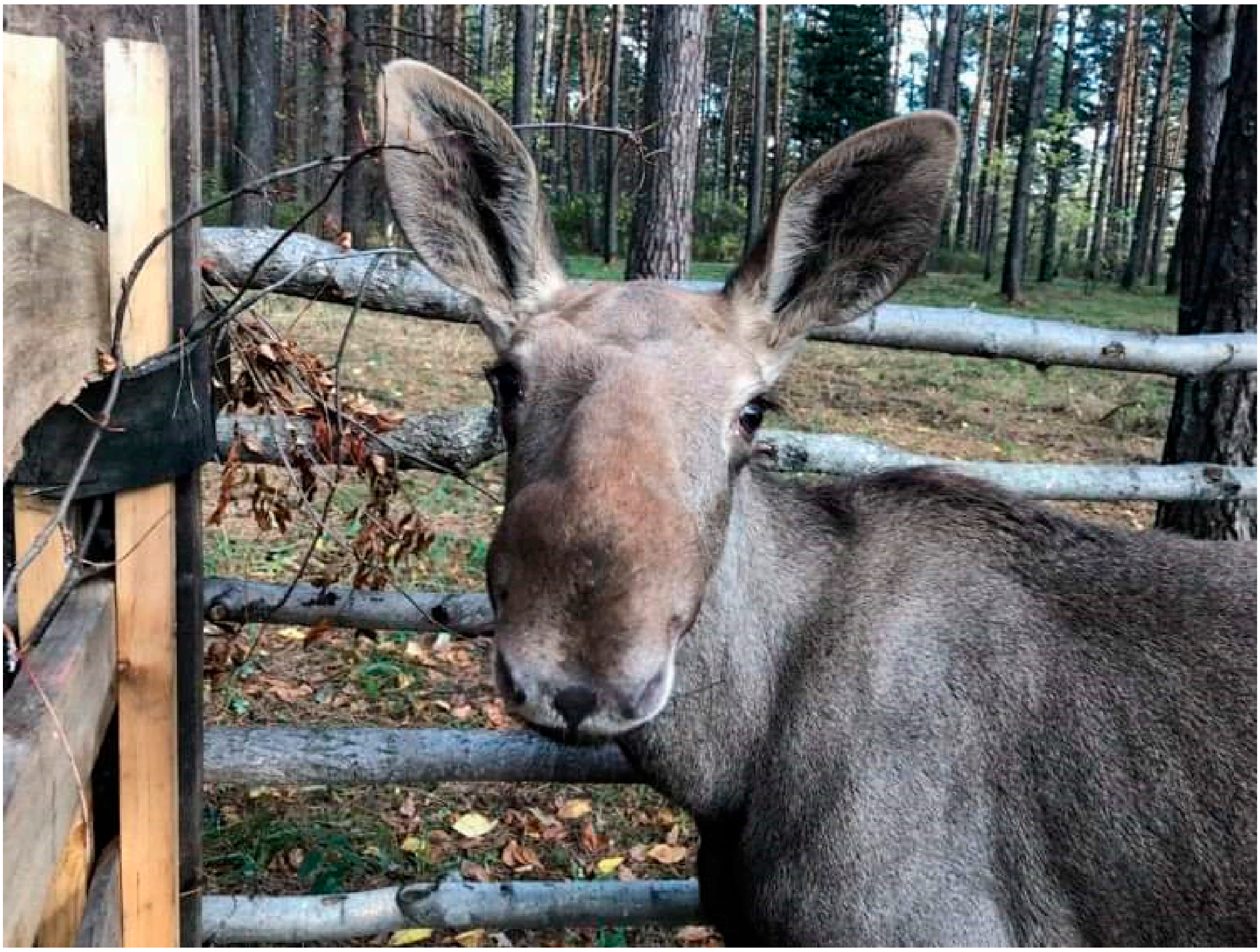

Figure S1. Photo of young elk contained in the rehabilitation center of the Tanay ski resort.

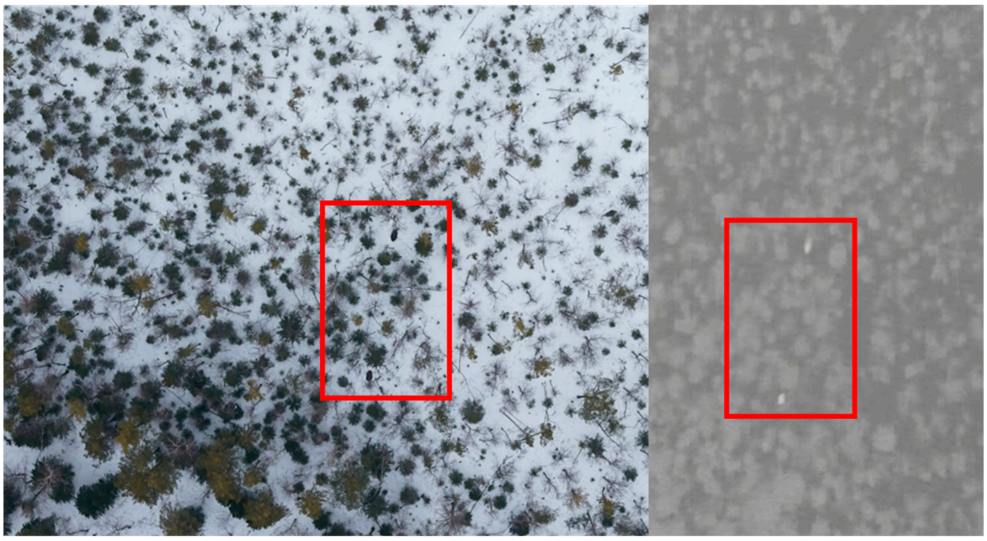

Figure S2. Photo (left) and thermal (right) imaging of the same area. The red frame marks the spot with thermal signatures of two elks.

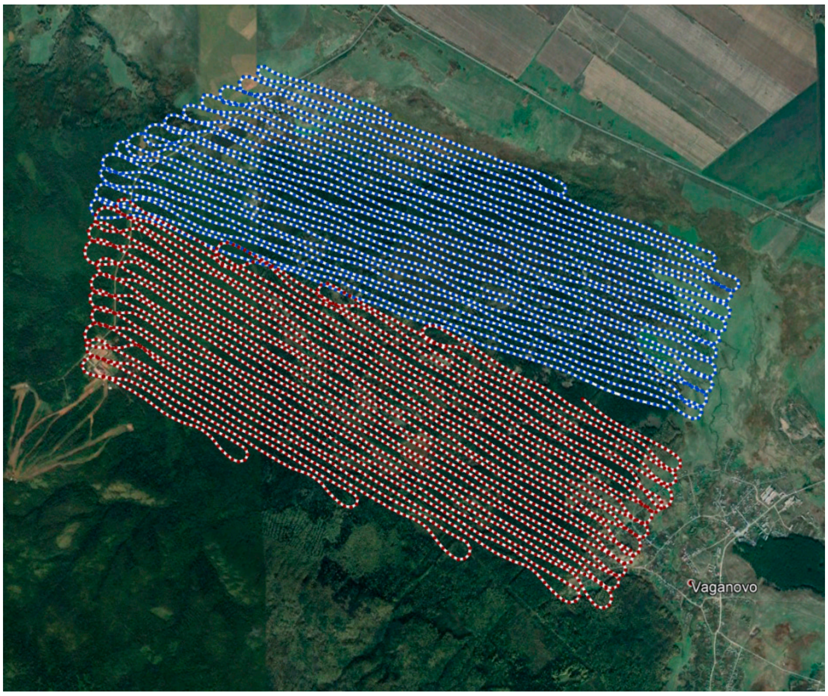

a

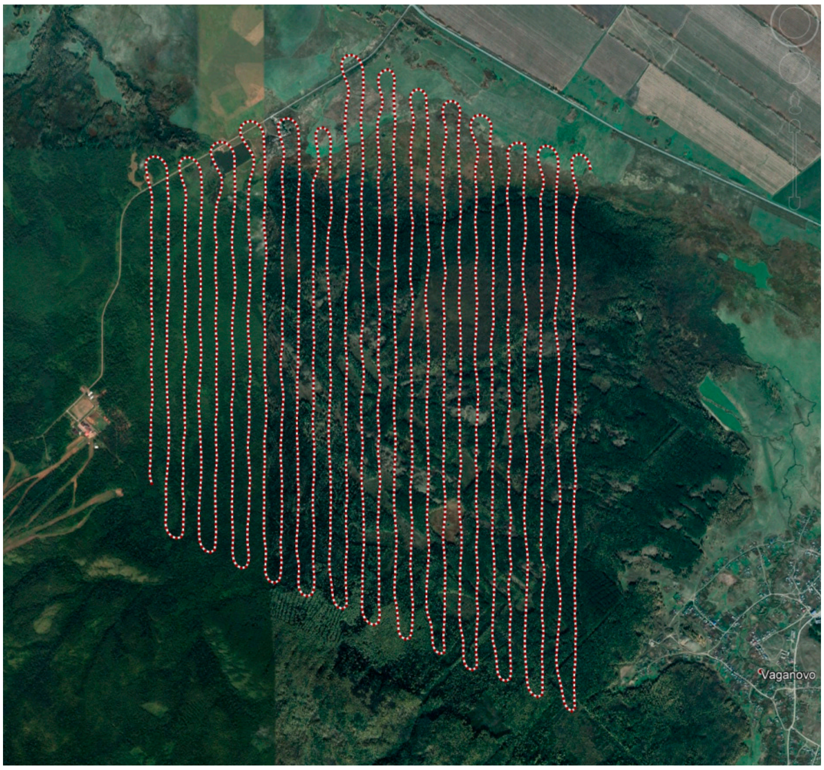

b

Figure S3. Flight routes in 2019 (a) and 2020 (b) on a Google map (Salair State Natural Park).

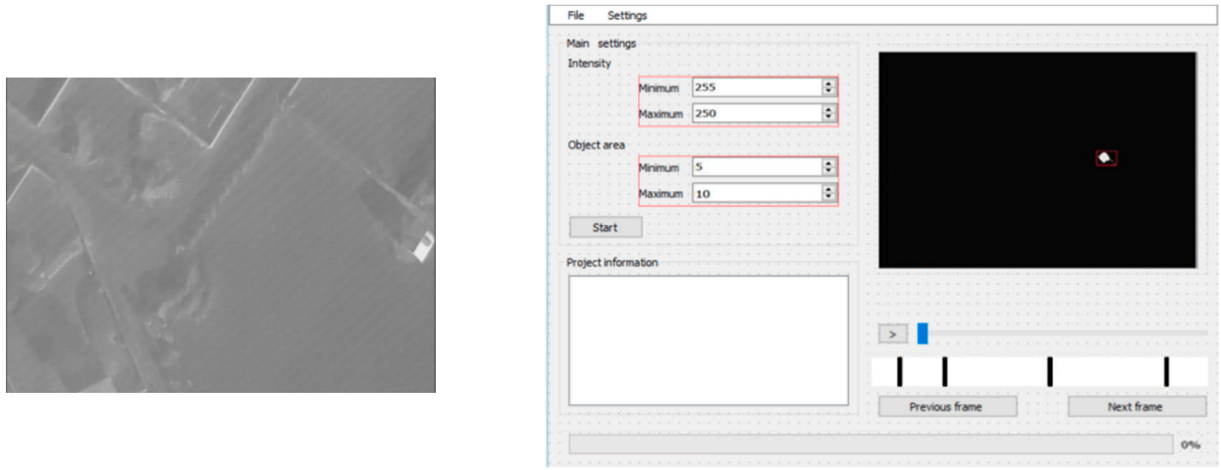

a

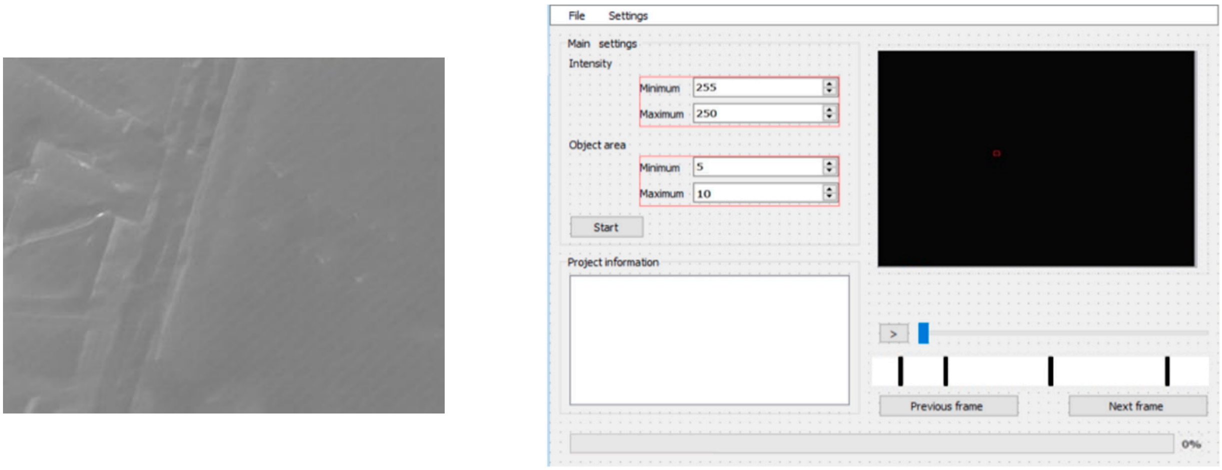

b

Figure S4. Infrared images processed by Thermal Infrared Object Finder: (a) height of exposure is 200 m, (b) height of exposure is 400 m.

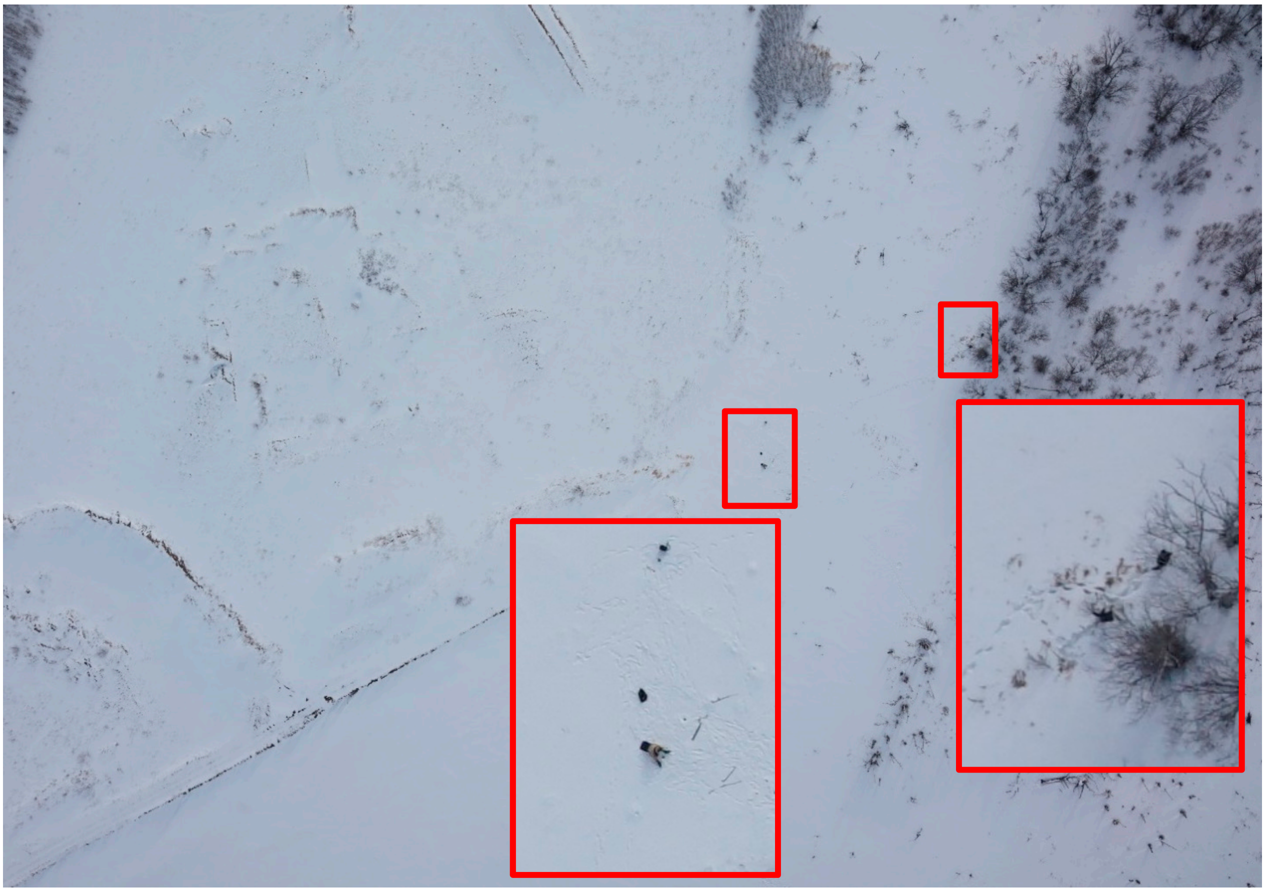
Figure S5. An example of untargeted objects (fishermen).

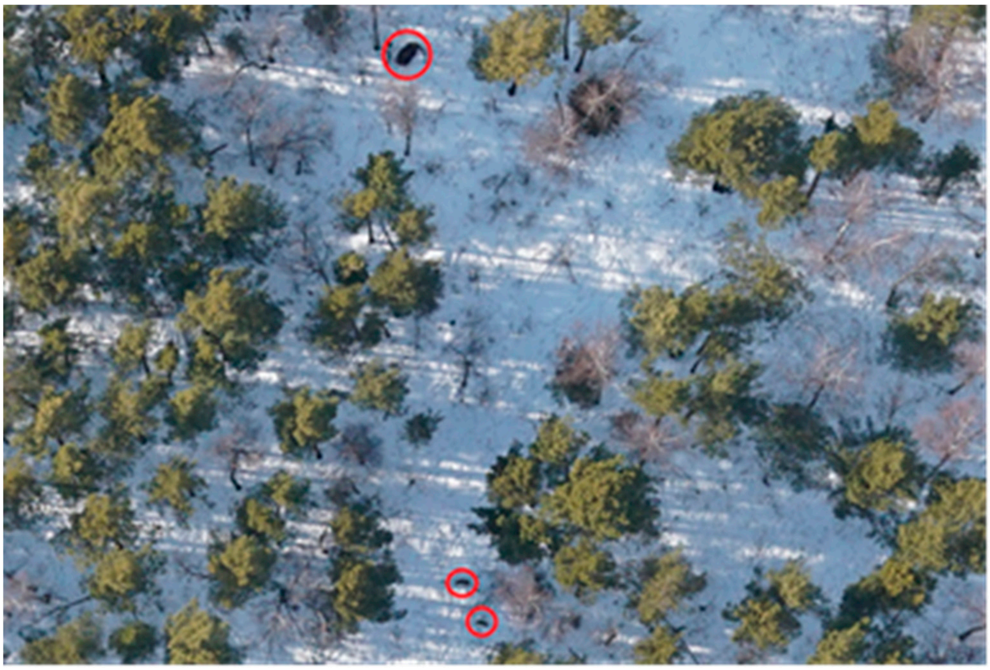

Figure S6. Snapshot where visual inspection revealed two wolves (red circles at the bottom) and an elk (red circle at the top).
